# Cross-talk between bone morphogenetic proteins and inflammatory pathways

**DOI:** 10.1186/s13075-015-0817-9

**Published:** 2015-11-23

**Authors:** Peter M. van der Kraan, Esmeralda N. Blaney Davidson

**Affiliations:** Experimental Rheumatology, Department of Rheumatology, Radboudumc, Geert Grooteplein 26, 6525 GA Nijmegen, The Netherlands

## Abstract

Pro-inflammatory cytokines and bone morphogenetic proteins are generally studied separately and considered to be elements of different worlds, immunology and developmental biology. Varas and colleagues report that these factors show cross-talk in rheumatoid arthritis synoviocytes. They show that pro-inflammatory cytokines not only stimulate the production of bone morphogenetic proteins but that these endogenously produced bone morphogenetic proteins interfere with the effects of pro-inflammatory cytokines on synoviocytes.

## Editorial

Varas and colleagues report in this volume that pro-inflammatory cytokines stimulate the production of bone morphogenetic proteins and that endogenously produced bone morphogenetic proteins interfere with the effects of pro-inflammatory cytokines on synoviocytes [1]. Both tumor necrosis factor (TNF)-α and interleukin (IL)-17 are hallmark cytokines with a dominant function in the immunology of rheumatoid arthritis (RA) [2, 3]. Targeting TNF-α is the prevailing biological therapy to treat RA patients, while targeting IL-17 could be a valuable addition for the management of a subgroup of patients [4]. Both TNF-α and IL-17 are factors that exemplify the dominant role of aberrant immune function in RA.

Bone morphogenetic proteins (BMPs), alternatively called body morphogenetic proteins, are ligands of the transforming growth factor (TGF)-β superfamily, which are central in embryology and developmental biology [5]. Ligands of this family determine cell and tissue fate from early embryogenesis to the formation of complex organs such heart, kidney and others [6, 7].

A few studies have addressed interactions between TNF-α/IL-17 and the BMPs. Expression of BMPs and regulation of their expression by pro-inflammatory cytokines has been shown in RA synovial tissue [8]. The expression of BMP-2 is enhanced by TNF-α in osteoarthritic chondrocytes and pro-inflammatory T-cell cytokines have been suggested to play a role in the differentiation of mesenchymal stromal cells into the osteoblast phenotype and in BMP-induced heterotopic ossification [9, 10]. These studies have focused on the induction of BMP expression by T-cell cytokines but have not investigated whether BMPs have a regulatory effect on T-cell cytokine function.

In a study published in this volume of *Arthritis Research & Therapy*, Varas and co-workers [1] investigated the modulatory role of BMPs on TNF-α and IL-17 responses in RA synoviocytes. What emerges from their study is that pro-inflammatory cytokines and BMP pathways interact. BMP receptors are expressed on RA synoviocytes, with ACTRIA/ALK2 and BMPRIA/ALK3 being the most abundant type I receptors while BMPRII was the most easily detectable type II receptor. In addition, BMP ligands, mainly BMP2, and both extracellular and intracellular BMP inhibitors were also expressed by RA synoviocytes. As shown by others, expression of BMP ligands was increased by IL-17 and TNF-α, showing an additive effect on BMP2, 6 and 7 expression. Interestingly, expression of the intracellular antagonists BAMBI and Smad7 was also enhanced by TNF-α/IL-17. However, Smad7 is generally not considered a genuine BMP signaling inhibitor but a blocker of TGF-β signaling.

The most appealing finding is that BMP ligands, produced by autocrine pathways, interfere with the effects of pro-inflammatory cytokines on RA synoviocytes. Blocking signaling of endogenously produced BMP ligands by DMH1, a specific BMP antagonist that inhibits signaling through ALK1, ALK2, and ALK3, enhanced both the mRNA and protein expression of IL-8 and granulocyte macrophage-colony stimulating factor, indicating that BMP ligands block the TNF-α/IL-17-induced production of these cytokines. Moreover, the expression of IL-17-induced CCL-2 and TNF-α/IL-17-induced matrix metalloproteinase (MMP)2 and MMP3 expression was further enhanced by inhibition of BMP signaling. Vice versa, addition of exogenous BMP6 inhibited the TNF-α/IL-17-induced elevated expression of these proteins.

This study shows that the presence of an endogenous BMP signaling pathway in RA synoviocytes meddles with the effects of pro-inflammatory cytokines on these cells. Since it can be anticipated that synoviocytes are exposed to TNF-α/IL-17 in a joint with active RA, activation of the BMP pathway might dampen the effects of these pro-inflammatory cytokines. Furthermore, the authors hypothesize that BMP signaling could have an anti-inflammatory role in the control and maintenance of low levels of pro-inflammatory factors in healthy joints or the early stage of RA. In this way BMPs have a disease-controlling action. In contrast, BMPs upregulate their own antagonists, primarily BAMBI and Smad7. In the case of Smad7 it can be anticipated that mainly TGF-β signaling via the Smad2/3-Smad4 route is blocked. Due to the potent and extensive, chiefly anti-inflammatory, action of TGF-β, one would expect that BMP-related induction of Smad7 will limit the anti-inflammatory action of TGF-β. In this way induction of BMP and TGF-β inhibitors could contribute to the chronic inflammation seen in RA. What is more, the use of BMPs as an anti-inflammatory route does not seem to be an attractive option to follow due to the upregulation of BMP and TGF-β signaling inhibitors that will interfere with the potential anti-inflammatory action of these ligands.

A limitation of the study by Varas and colleagues is that synoviocytes between passages 4 and 9 were used. Although it is known that RA synoviocytes keep their phenotype in vitro, it cannot be excluded that the role of BMP signaling is altered during culture on plastic. Moreover, data are not provided that show whether differences exist between different patient (subgroups) nor what the effect of medication is. However, due to the late passage in which cells are studied, the influence of medication can largely be ruled out.

The major value of this paper is that it shows that BMP and inflammatory pathways interact. Although these pathways, and factors involved, are in many cases studied separately, Mother Nature is not confined to man-made boxes. More and more it becomes clear that human physiology is an holistic system in which the same factors are used over and over and which shows interference on all levels. The paper of Varas and colleagues clearly shows that inflammation and tissue development and maintenance are not separate systems but instead are integrated.

## Abbreviations

BMP: Bone morphogenetic protein
IL: Interleukin
MMP: Matrix metalloproteinase
RA: Rheumatoid arthritis
TGF: Transforming growth factor
TNF: Tumor necrosis factor

## References

[1] Varas A, Valencia J, Lavocat F, Martínez VG, Thiam NN, Hidalgo L (2015). Blockade of bone morphogenetic protein signaling potentiates the pro-inflammatory phenotype induced by interleukin-17 and tumor necrosis factor-α combination in rheumatoid synoviocytes. Arthritis Res Ther.

[2] Brennan FM, Maini RN, Feldmann M (1992). TNF alpha—a pivotal role in rheumatoid arthritis?. Br J Rheumatol.

[3] van den Berg WB, Miossec P (2009). IL-17 as a future therapeutic target for rheumatoid arthritis. Nat Rev Rheumatol.

[4] Roeleveld DM, Koenders MI (2015). The role of the Th17 cytokines IL-17 and IL-22 in rheumatoid arthritis pathogenesis and developments in cytokine immunotherapy. Cytokine.

[5] Bier E, De Robertis EM (2015). BMP gradients: a paradigm for morphogen-mediated developmental patterning. Science.

[6] Jain R, Li D, Gupta M, Manderfield LJ, Ifkovits JL, Wang Q (2015). Integration of Bmp and Wnt signaling by Hopx specifies commitment of cardiomyoblasts. Science.

[7] Nishinakamura R, Sakaguchi M (2014). BMP signaling and its modifiers in kidney development. Pediatric Nephrol.

[8] Lories RJ, Derese I, Ceuppens JL, Luyten FP (2003). Bone morphogenetic proteins 2 and 6, expressed in arthritic synovium, are regulated by proinflammatory cytokines and differentially modulate fibroblast-like synoviocyte apoptosis. Arthritis Rheum.

[9] Fukui N, Zhu Y, Maloney WJ, Clohisy J, Sandell LJ (2003). Stimulation of BMP-2 expression by pro-inflammatory cytokines IL-1 and TNF-alpha in normal and osteoarthritic chondrocytes. J Bone Joint Surg Am.

[10] Rifas L (2006). T-cell cytokine induction of BMP-2 regulates human mesenchymal stromal cell differentiation and mineralization. J Cell Biochem.

